# *Clostridium perfringens* Virulent Bacteriophage CPS2 and Its Thermostable Endolysin LysCPS2

**DOI:** 10.3390/v10050251

**Published:** 2018-05-11

**Authors:** Eunsu Ha, Bokyung Son, Sangryeol Ryu

**Affiliations:** 1 Department of Food and Animal Biotechnology, Seoul National University, Seoul 08826, Korea; esha0521@gmail.com (E.H.); sonbk0722@gmail.com (B.S.); 2 Department of Agricultural Biotechnology, and Research Institute of Agriculture and Life Sciences, Seoul National University, Seoul 08826, Korea

**Keywords:** bacteriophage, endolysin, *Clostridium perfringens*

## Abstract

*Clostridium perfringens* is one of the most common causes of food-borne illness. The increasing prevalence of multidrug-resistant bacteria requires the development of alternatives to typical antimicrobial treatments. Here, we isolated and characterized a *C. perfringens*-specific virulent bacteriophage CPS2 from chicken feces. The CPS2 phage contains a 17,961 bp double-stranded DNA genome with 25 putative ORFs, and belongs to the *Picovirinae*, subfamily of *Podoviridae*. Bioinformatic analysis of the CPS2 genome revealed a putative endolysin, LysCPS2, which is homologous to the endolysin of *Clostridium* phage phiZP2 and phiCP7R. The enzyme showed strong lytic activity against *C. perfringens* with optimum conditions at pH 7.5–10, 25–65 °C, and over a broad range of NaCl concentrations. Interestingly, LysCPS2 was found to be highly thermostable, with up to 30% of its lytic activity remaining after 10 min of incubation at 95 °C. The cell wall binding domain in the C-terminal region of LysCPS2 showed a binding spectrum specific to *C. perfringens* strains. This is the first report to characterize highly thermostable endolysin isolated from virulent *C. perfringens* bacteriophage. The enzyme can be used as an alternative biocontrol and detection agent against *C. perfringens*.

## 1. Introduction

*Clostridium perfringens* is a Gram-positive anaerobic bacterium that has the ability to form spores [1]. *C. perfringens* is responsible for a wide range of diseases: including gas gangrene (clostridial myonecrosis), necrotic enteritis, and non-foodborne gastrointestinal infections [2]. Approximately 5% of all *C. perfringens* strains produce *C. perfringens* enterotoxin (CPE), which causes diarrhea and abdominal cramping symptoms. Most CPE-positive stains are classified as type A, and food poisoning by *cpe*-producing *C. perfringens* type A is the second most common foodborne illness in developed countries [3]. In addition, *C. perfringens* has become a significant problem in the poultry industry because it is a causative agent of necrotic enteritis, characterized by outbreaks with high mortality and small intestinal mucosal necrosis [4]. Increased mortality, economic loss, and contamination of poultry products for human consumption are important concerns regarding *C. perfringens* in the poultry industry.

Furthermore, the increasing incidence of antibiotic resistance of bacterial pathogens and the lack of novel antibiotics have become serious worldwide problems. Bacteriophages (phages) and gene products such as endolysins have been attracting considerable attention as alternatives to antibiotics. Endolysins are phage-encoded peptidoglycan hydrolases that break down the bacterial peptidoglycan at the end of their reproduction cycles to release the viral progeny [5]. The purified endolysin protein has potent hydrolytic activity against Gram-positive bacteria when applied exogenously. In addition, endolysins have significant advantages over classic antibiotics, such as narrow host specificity, high sensitivity, and low probability for development of resistant bacteria [6].

To date, there have been few reports on *C*. *perfringens* phage endolysins. Most studies have attempted to isolate endolysin from the prophage because of the relative difficulties in isolating the phage from anaerobic *C. perfringens*. *N*-acetylmuramidases of *C. perfringens* phage phiSM101 and *N*-acetylmuramoyl-l-alanine amidase of φ3626 phage were isolated from lysogenic *C. perfringens* strains and characterized [7,8]. CP25L endolysin was examined for the activity to kill *C. perfringens*, and endolysin delivery by *Lactobacillus johnsonii* was reported [9]. Two endolysins from the clostridial phages ΦCP39O and ΦCP26F have been reported to have lytic activity against *C. perfringens* stains [10]. However, detailed information about these endolysins have not been reported.

Here we isolated novel virulent *C. perfringens* bacteriophage CPS2 from chicken feces, and its predicted endolysin LysCPS2 was identified in the genome of bacteriophage CPS2. The gene was cloned and expressed in *Escherichia coli*, and the purified endolysin was biochemically characterized for its potential as an antimicrobial and a detection agent.

## 2. Materials and Methods

### 2.1. Bacterial Strains, and Growth Conditions

*C. perfringens* ATCC 13124 was used as a host strain for isolation and propagation of the bacteriophage CPS2. *E. coli* BL21 was grown in Luria-Bertani (LB) broth (Difco, Detroit, MI, USA) at 37 °C and used as the host for expression of the recombinant LysCPS2. Bacterial strains that were used for antimicrobial spectrum determination are listed in Table 1, along with the results. All of the bacterial strains were routinely grown at 37 °C in Brain Heart Infusion (BHI) broth medium (Difco) under anaerobic conditions.

### 2.2. Isolation and Propagation of Bacteriophage CPS2

To isolate a bacteriophage, we applied the same method as described in the previous study [11]. Isolated phages were amplified by serial propagation and concentrated by polyethylene glycol precipitation and subsequent CsCl density gradient ultracentrifugation (78,500× *g* at 4 °C for 2 h). The concentrated phages were dialyzed using 2 L of standard dialysis buffer (10 mM NaCl, 10 mM MgSO_4_ and 1 M Tris-HCl; pH 8.0) for 2 h. The phage stock obtained was stored in glass vials at 4 °C.

### 2.3. Transmission Electron Microscopy (TEM) Analysis

Purified CPS2 (1 × 10^9^ PFU/mL) was placed on carbon-coated copper grids and negatively stained with 2% aqueous uranyl acetate (pH 4.0) for 20 s. The morphology of CPS2 was analyzed by TEM (LEO 912AB transmission electron microscope; Carl Zeiss, Wezlar, Germany). Images were scanned at the National Academy of Agricultural Science (Jeonju, South Korea).

### 2.4. DNA Purification and Whole Genome Sequencing of Bacteriophage CPS2

To extract genomic DNA from CPS2, host DNA was removed by treatment of the virions with DNaseI and RNaseA (1 μg/mL each) at room temperature for 30 min. The virions were then lysed by reacting with proteinase K mixture (50 μg/mL proteinase K, 20 mM ethylenediaminetetraacetic acid (EDTA), 0.5% sodium dodecyl sulfate (SDS)) at 56 °C for 1 h. After lysis, the DNA was purified by the phenol-chloroform extraction [12] and concentrated by ethanol precipitation [13]. The purified genomic DNA of CPS2 was sequenced using the GS-FLX Titanium sequencer (Roche Holding AG, Basel, Switzerland). Total 23,050 sequencing reads obtained were assembled using the GS De Novo Assembler version 2.9 (Roche Holding AG, Basel, Switzerland) with default parameters. Additional DNA sequencing of phage was performed to identify end regions of genomic DNA sequence using primers (CPS2endF, 5’-CAC CCT GGA GCA TTT ACA C-3’; CPS2endR, 5’-TCC ATA ACA GAC AAT AAA AAT TTT AAA T-3’) in Macrogen inc. (Seoul, South Korea). The position of open reading frames (ORFs) was predicted by bioinformatics tools, including Glimmer 3.02 [14] and Rapid Annotation using Subsystem Technology (RAST) software [15]. The function of each ORF was predicted using NCBI BLASTP and InterProScan [14] databases. Based on the information, each ORF’s name was annotated manually. The gene encoding endolysin was identified, and its domain structure was investigated using InterProScan databases. The complete genome sequence of CPS2 phage was deposited in GenBank under accession number MH248069.

### 2.5. Cloning, Expression, and Purification of LysCPS2

The endolysin gene (*lysCPS2*) was amplified from the genomic DNA of the bacteriophage CPS2 by polymerase chain reaction (PCR) using primers lysCPS2F (5’-GCG GGA TCC ATG AAA ATA ATA CAA TCA AAT ATC CAT TTT-3’) and lysCPS2R (5’-CGC AAG CTT TTA GTC TTT TTT AAT ATA TTT TGC GGA-3’). The PCR product was cloned into pET28a (Novagen, Madison, WI, USA), which has an *N*-terminal hexahistidine (His)-tag sequence. Plasmid with a correct insert was transformed into competent *E. coli* BL21 (DE3). Expression of the recombinant LysCPS2 was induced with 0.5 mM isopropyl-b-d-thiogalactopyranoside, with adjusted OD600 to 0.6–0.8, followed by incubation for an additional 15 h at 30 °C. Bacterial cells were suspended in lysis buffer (50 mM Tris-HCl, 100 mM sodium chloride, pH 7.5) and disrupted by sonication (Branson Ultrasonics, Shanghai, China). After centrifugation at 15,000× *g* for 40 min, the supernatant was collected, mixed with 500 μL of nickel-nitrilotriacetic acid (Ni-NTA) agarose (Qiagen, Hilden, Germany) and incubated at 4 ℃ for 1 h with gentle shaking. The flow-through was discarded and the resin was serially washed with 10 mL of 10 mM imidazole and 5 mL of 20 mM imidazole. An elution buffer (50 mM Tris-HCl, 100 mM sodium chloride, and 250 mM imidazole; pH 7.5) was used to elute the protein. The purified protein was stored at −4 °C until use, after the buffer was changed to the storage buffer (50 mM Tris-HCl, 200 mM sodium chloride, pH 7.5, 30% glycerol) using PD Miditrap G-25 (GE Healthcare, Little Chalfont, UK).

### 2.6. Lytic Activity Assay

The lytic activity of the endolysin against bacterial cells was assayed by monitoring the decrease in OD_600_. All tested bacteria were cultivated to the exponential phase. Cells were harvested and resuspended with reaction buffer (50 mM Tris-HCl, 200 mM NaCl, pH 7.5) to OD_600_ to 0.8–1.0. The endolysin (50 μL) was added to the cell suspension (950 μL), followed by incubation at room temperature, unless indicated otherwise. OD_600_ values were monitored over time. The lytic activity was calculated after 40 min as follows: {ΔOD_600_ test (endolysin added)-ΔOD_600_ control (buffer only)}/initial OD_600_. Antimicrobial spectrum was tested by plate lysis assay as previously described [16]. In brief, 10 μL of diluted endolysin in reaction buffer (32 μM; final concentration) was spotted onto a freshly prepared bacterial lawn on BHI agar plates. Spotted plates were air-dried in a laminar flow hood for 15 min and incubated overnight at 37 °C. To evaluate the effect of pH on LysCPS2 enzymatic activity, the endolysin (162 nM) was added to *C. perfringens* cells suspended with a universal pH buffer [17]. The universal buffer consists of 50 mM KCl, 10 mM KH_2_PO_4_, 10 mM Na-citrate, and 10 mM H_3_BO_4_, and was adjusted to different pH values—between 4 and 10—using 5 M NaOH or 5 M HCl. Different temperatures (25–95 °C) were applied to test the effect of temperature on LysCPS2 (162 nM) enzymatic activity. To evaluate the stability of the endolysin, the lysis assays were performed against *C. perfringens* ATCC 13124 at room temperature and pH 7.5 after the enzyme was incubated for 10 min at different temperatures. The influence of NaCl on lytic activity of LysCPS2 (162 nM) was tested with the addition of concentrations of 0, 100, 200, 300, 400, and 500 mM NaCl. The effects of metal ions on lysis activity were determined as previously reported [6]. To chelate metal ions attached to the endolysin, thereby inhibiting its catalytic function, EDTA (100 mM; final concentration) was added to the endolysin (4.86 μM) and incubated at 37 °C for 1 h. The EDTA was removed by exchanging the endolysin into the reaction buffer using a PD trap G-25 column. The EDTA-treated enzyme was added to cell suspensions with metal ions (MgCl_2_, CaCl_2_, MnCl_2_, ZnCl_2_, CuCl_2_; 1.0 mM final concentration), and the lysis activity was assayed in the reaction buffer.

### 2.7. CBD Binding and Fluorescence Microscopy

The gene encoding the putative CBD (between bases 457–681 of *lysCPS2*) was amplified from the genomic DNA of CPS2 by PCR using primers lysCPS2_CBDF (5’-GCG GGA TCC GGA AAC TTA GAT TTA AAC AAA TTA AGA ACA GAT GTA AAC-3’) and lysCPS2_CBDR (5’-CGC AAG CTT TTA GTC TTT TTT AAT ATA TTT TGC GGA-3’). The resulting PCR product was cloned using the BamHI and SalI restriction sites into pET28a-mCherry (*mCherry* gene inserted between NdeI and BamHI sites into pET28a) [18]. mCherry-tagged LysCPS2_CBD protein was produced in *E. coli* BL21 and purified by affinity chromatography, as described earlier [19]. Purified protein at a final concentration of 100 μM was added to *C. perfringens* cells, and the mixture was then incubated for 10 min at 25 °C. Subsequently, cells were collected by centrifugation and washed twice with Dulbecco’s phosphate-buffered saline (PBS). For fluorescence microscopy, images were captured on a DE/Axio Imager A1 microscope (Carl Zeiss) with a charge-coupled-device camera using AxioVision release 4.7 (Carl Zeiss, Oberkochen, Germany).

## 3. Results and Discussion

### 3.1. Morphology of Phage CPS2

**Figure 1:**
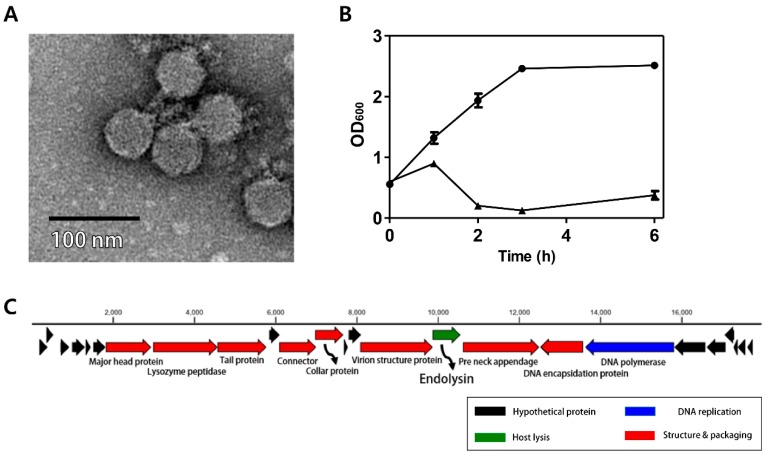
Characterization of *C. perfringens* virulent phage CPS2. (**A**) TEM image of CPS2; (**B**) bacterial challenge assay of CPS2 against *C. perfringens* ATCC 13124. The closed circle indicates non-phage-treated *C. perfringens* ATCC 13124, and the closed triangle indicates phage-treated *C. perfringens* ATCC 13124; (**C**) genome map of CPS2. Red: structure and packaging, blue: DNA replication, green: host lysis, black: hypothetical protein. Twenty-five putative ORFs were predicted in the CPS2 genome.

The *C. perfringens* phage CPS2 was isolated from chicken feces using *C. perfringens* isolate as a host stain. Morphological observation of phage CPS2 revealed that it belongs to the family *Podoviridae* due to the presence of short and non-contractile tails (Figure 1A). The diameter of the icosahedral head was approximately 40 nm and the length of non-contractile tails (from baseplate of virion to tail fiber) was 15 nm.

### 3.2. Antibacterial Properties of Phage CPS2

The bacterial challenge assay was performed to evaluate the growth inhibition ability of the CPS2 phage. When phage CPS2 was added to exponentially growing *C. perfringens* at a multiplicity of infection (MOI) of 1.0, complete inhibition of host cells was observed 1 h after phage infection, and growth inhibition was maintained for up to 6 h (Figure 1B). A host range test showed that CPS2 can inhibit the growth of only *C. perfringens* ATCC 13124, and *C. perfringens* human stool isolate 3 out of the 10 tested *C. perfringens* strains. CPS2 phage did not show lytic activity against other clostridial bacteria or other Gram-positive bacteria, indicating very narrow host specificity (Table 1).

### 3.3. Genomic Analysis of CPS2

The complete genome of *C. perfringens* bacteriophage CPS2 comprises 17,961-bp with an overall G + C content of 33.30%. Twenty-five putative ORFs were identified, and no tRNA genes were detected. The functional ORFs were clustered into 4 functional groups of DNA replication, host lysis, structure plus packaging, and additional function (Figure 1C). The sequence analysis showed the linear structure of CPS2 double-stranded DNA, as well as the inverted terminal repeats (ITR) of 366 nucleotide pairs on the phage genomic termini. Furthermore, the CPS2 genome encoded a putative Type-B DNA polymerase that is utilized in a protein-primed replication mechanism, implying the presence of terminal proteins for DNA replication [22]. These findings suggest that CPS2 belong to the *Podoviridae* sub-family *Picovirinae* [23]. Importantly, the toxin production- and bacterial virulence-associated genes were not identified in the CPS2 genome. Genes associated with lysogenization were not detected, suggesting that CPS2 is a virulent phage.

### 3.4. Identification and Expression of the LysCPS2 Endolysin

**Figure 2:**
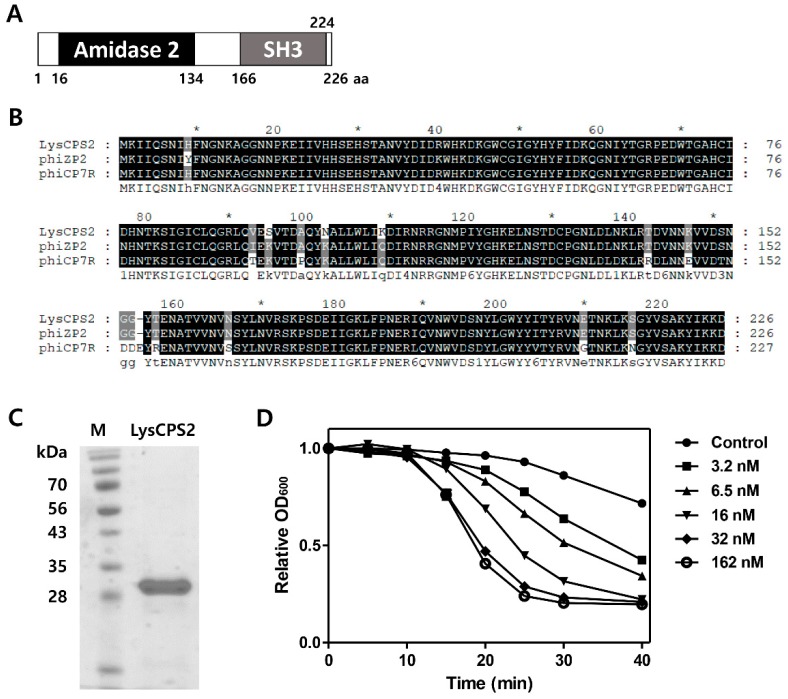
Modular structure and lytic activities of the LysCPS2 endolysin from CPS2. (**A**) Schematic representation of LysCPS2. The conserved amidase2 domain is shown; (**B**) Sequence alignment of various Clostridial phage endolysins; CPS2 phage endolysin, phiZP2 phage endolysin, phiCP7R phage endolysin; (**C**) SDS-PAGE analysis of purified LysCPS2. M: standard molecular weight marker, LysCPS2: purified LysCPS2 fraction; (**D**) Lysis of *C. perfringens* ATCC 13124 cells treated externally with various concentrations of recombinant LysCPS2. Optical density was measured periodically after LysCPS2 treatment.

The putative endolysin gene was identified from the CPS2 genome and designated as LysCPS2. Pfam and Conserved Domain Database analysis revealed that LysCPS2 is a putative *N*-acetylmuramoyl-l-alanine amidase that consists of N-terminal amidase_2 domain (PF01520) as an enzymatically active domain (EAD) and a C-terminal SH3_3 (PF08239) as a cell wall binding domain (CBD). Amino acid sequence alignment revealed that LysCPS2 was highly homologous to endolysins of *Clostridium* phage phiZP2 and phiCP7R at the amino acid sequence level (Figure 2B) [22] The N-terminal region from LysCPS2 showed 51% sequence identity with previously reported *Clostridium* phage vB_CpeS-CP51 endolysin. Although BLAST analysis revealed many proteins homologous to LysCPS2, most of them were autolysins. The LysCPS2 was cloned and expressed in *E. coli* with an N-terminal His-tag. Sodium dodecyl sulfate polyacrylamide gel electrophoresis (SDS-PAGE) showed a single band of the purified endolysin (Figure 2C). Turbidity reduction assay revealed that 32 nM of the purified LysCPS2 showed saturated lysis activity against *C. perfringens* ATCC 13124 cells (Figure 2D). These results indicate that LysCPS2 is highly active compared to endolysin from *C. difficile* phage ΦCD27, in which a similar amount of endolysin had barely exhibited lytic activity against *C. difficile* [24].

### 3.5. Antimicrobial Spectrum of LysCPS2

Antimicrobial activity against *Clostridium* and other Gram-positive bacterial strains was examined (Table 1). All of the tested *C. perfringens* strains were susceptible to LysCPS2, indicating a much broader antimicrobial spectrum of LysCPS2, considering the narrow host range of the CPS2 phage. However, other *Clostridium* species such as *C. histolyticum* and *C. indolis* were not lysed by treatment of LysCPS2, meaning that its enzymatic activity requires species-specific moieties of the cell wall component. This enzyme did not show lytic activity against other Gram-positive bacteria, such as *Bacillus cereus, Bacillus subtilis, Listeria monocytogenes,* and *Staphylococcus aureus. B. cereus*, *B. subtilis,* and *L. monocytogenes* have an A1γ type peptidoglycan directly cross-linked with *meso*-diaminopimelic acid (m-DAP), and *S. aureus* has an A3α type cross-linked by penta-glycine bridges in its peptidoglycan. *C. perfringens* has a different type of peptidoglycan structure containing L,L-DAP and glycine instead of m-DAP in the A1γ type peptidoglycan [25].

### 3.6. pH, Temperature, NaCl, and Metal Effects on LysCPS2 Activity

**Figure 3:**
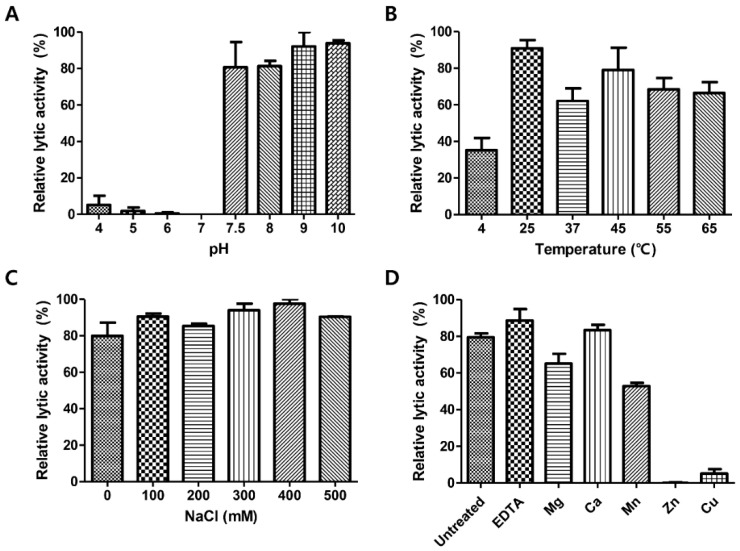
Effects of pH, temperature, NaCl and metal ions on the lytic activity of LysCPS2. Effects of (**A**) pH, (**B**) temperatures, (**C**) NaCl, and (**D**) metal ions on the lytic activity of LysCPS2 against *C. perfringens* ATCC 13124 cells. Each column represents the mean of triplicate experiments, and error bars indicate the standard deviation.

To determine optimum conditions for the endolysin activity, the biochemical properties of LysCPS2 were evaluated. Analysis of lytic activity at different pH levels showed that LysCPS2 had the highest lytic activity at pH 7.5–10 (Figure 3A). More than 60% residual lytic activity was observed in the temperature range from 25 °C to 65 °C (Figure 3B). The effect of ionic strength on the lytic activity of LysCPS2 was assessed at various concentrations of NaCl, ranging from 0 to 500 mM (Figure 3C). LysCPS2 retained its lytic activity even at 500 mM NaCl, suggesting that LysCPS2 is highly stable in a broad range of NaCl concentrations in contrast to the *C. perfringens* phage phiSM101 endolysin that showed reduced activity in the presence of more than 200 mM of NaCl [7]. Even though phiSM101 endolysin showed 36% amino acid sequence identity with LysCPS2, it was the only *C. perfringens* endolysin that could be compared with the influence of NaCl on the lytic activity of LysCPS2. To examine the effect of metal ions on the enzyme activity of LysCPS2, metal ions were initially removed from the endolysin with 100 mM EDTA. Addition of EDTA showed little effect on the LysCPS2 activity, indicating that LysCPS2 activity is independent of the presence of divalent cations. All tested metal ions except Ca^2+^ decreased enzymatic activity of LysCPS2, and the enzyme was completely inactivated in the presence of Zn^2+^ (Figure 3D). Although many divalent ions have been reported to enhance the enzymatic activity of endolysin, the inhibition effect by certain metal ions on endolysin activity has also been reported in several studies [26,27]. The peptidoglycan hydrolase of *Burkholderia pseudomallei* phage ST79 showed reduced lytic activity in the presence of Zn^2+^, Mg^2+^, or Mn^2+^ [28]. The inhibition effect of Zn^2+^ is also found in the T5 phage endolysin [29].

### 3.7. Determination of Thermal Stability

**Figure 4:**
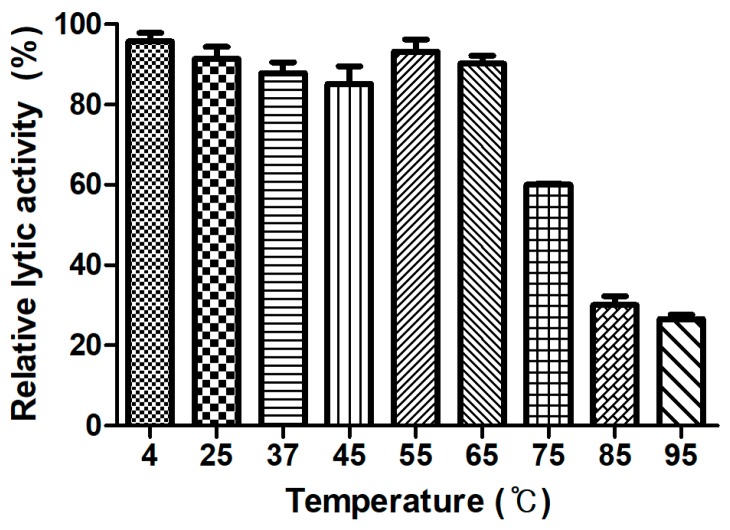
LysCPS2 thermal stability. LysCPS2 was incubated at different temperatures for 10 min, then cooled on ice before enzyme assay at 25 °C. Relative lytic activities are calculated using the activity of enzyme stored at 4 °C, which showed the maximal activity.

Generally, endolysins are not heat stable, and most lose their hydrolase activity above 50–60 °C [30]. However, few endolysins such as listerial phage endolysins HPL511, HPL118, and HPLP35 have been reported to be thermostable, showing residual activity after 10 min incubation at 90 °C [31]. *Stenotrophomonas maltophilia* phage endolysin P28 retained 55% of its activity after treatment at 70 °C for 30 min [32], and a thermostable chimeric endolysin, PlyGVE2CpCWB, targeting *C. perfringens* has exhibited 57% residual activity at 55 °C [33]. LysCPS2 showed remarkable heat stability. The lytic activity after pre-incubation of the enzyme at temperatures between 4 °C and 65 °C was not reduced compared with the heat-untreated control. LysCPS2 retained more than 30% of its activity after heating at 95 °C for 10 min and 60% after heating at 75 °C for 10 min (Figure 4). This is the first report to characterize highly thermostable endolysin isolated from virulent *C. perfringens* bacteriophage. This enzyme will provide powerful tools for many applications in molecular biology, biotechnology, and medicine [34].

### 3.8. Binding Activity of LysCPS2_CBD

**Figure 5:**
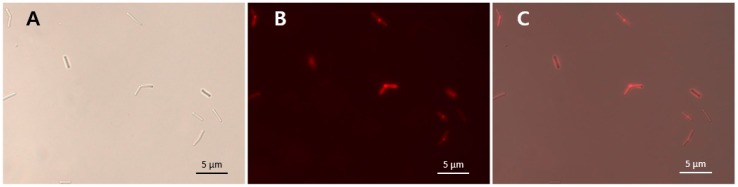
mCherry_LysCPS2_CBD binding activity. LysCPS2_CBD binds to *C. perfringens* human isolate cells. Bright field (**A**), fluorescence (**B**) and merged (**C**) images are shown.

The specific bacterial binding activity of the putative LysCPS2_CBD was identified with an mCherry (red fluorescent protein) fusion protein (Figure 5). As shown in Table 1, all tested *C. perfringens* cells were decorated by mCherry_LysCPS2_CBD, whereas other *Clostridium* species and other Gram-positive bacteria could not be labeled by the fusion protein (Table 1). These results demonstrated that LysCPS2_CBD specifically binds to *C. perfringens* cells, which is consistent with the antimicrobial spectrum of the LysCPS2. The specificity and affinity of LysCPS2_CBD could be useful for developing an efficient bio-probe against *C. perfringens*.

## 4. Conclusions

In this study, we isolated a *C. perfringens* virulent phage CPS2 and identified a gene encoding LysCPS2 endolysin from CPS2 genome. LysCPS2 is a highly thermostable endolysin showing 30% of its lytic activity against *C. perfringens* even after 10 min of incubation at 95 °C. Optimum conditions for LysCPS2 were at pH 7.5–10, 25–65 °C, and over a broad range of NaCl concentrations, suggesting that LysCPS2 has high potential as an effective antibacterial agent to control *C. perfringens*. The cell wall binding domain in the C-terminal region of LysCPS2 showed a binding spectrum specific to *C. perfringens* strains. Taken together, LysCPS2 would be useful for the development of a powerful biocontrol and detection agent against *C. perfringens.*

## Figures and Tables

**Table 1:** Antimicrobial spectra of the CPS2 and LysCPS2, and binding spectrum of LysCPS2_CBD.

Bacterial Strain	CPS2 Plaque Formation	Lysis Zone Formation by LysCPS2	Binding Activity of LysCPS2_CBD	Reference or Source
***Clostridium* strains**				
*C. perfringens* H3	Lysis from without	+	+	[20]
*C. perfringens* ATCC 3624	Lysis from without	+	+	ATCC ^a^
*C. perfringens* ATCC 13124	+	+	+	ATCC
*C. perfringens* FORC25	−	+	+	This study
*C. perfringens* human stool isolate 1	−	+	+	This study
*C. perfringens* human stool isolate 2	−	+	+	This study
*C. perfringens* human stool isolate 3	+	+	+	This study
*C. perfringens* human stool isolate 4	Lysis from without	+	+	This study
*C. histolyticum* ATCC 19401	−	−	−	ATCC
*C. indolis* ATCC 25771	−	−	−	ATCC
**Other Gram-positive bacteria**				
*Bacillus cereus* ATCC 10987	−	−	−	ATCC
*Bacillus subilis* ATCC 23857	−	−	−	ATCC
*Listeria monocytogenes* EGD-e	−	−	−	[21]
*Staphylococcus aureus* RN4220	−	−	−	[16]

^a^ ATCC, American Type Culture Collection; +, positive activity; −, negative activity.
